# Data for crystallisation of a homologous series of single and mixed n-alkanes (C_16_ – C_23_) from representative hydrocarbon fuel solvents

**DOI:** 10.1016/j.dib.2023.109198

**Published:** 2023-05-02

**Authors:** Alexander S.M. Jackson, Dhanesh Goberdhan, Peter J. Dowding, Kevin J. Roberts

**Affiliations:** a School of Chemical and Process Engineering, University of Leeds, Leeds LS2 9JT, United Kingdom; b Infineum UK Ltd, Milton Hill Business and Technology Centre, Abingdon OX13 6BB, United Kingdom

**Keywords:** Solution solubility and ideality, n-Alkanes, Hydrocarbon fuels, Nucleation mechanism and kinetics

## Abstract

The data presented in this article relates to the crystallisation of 8 single n-alkanes, C_16_H_34_ – C_23_H_48_ in representative diesel solvents dodecane and toluene, as well as a mixture of these 8-alkanes with a composition representative of real diesel fuel in the same solvents. For the single alkane systems, the data was collected over a range of 5 concentrations ranging from 0.09 – 0.311x_i_, depending upon the system, and 4 concentrations for the 8-alkane mixture, 0.1 – 0.5x_i_. Raw average crystallisation and dissolution points as a function of cooling rate (q) from a polythermal methodology are presented. Along with the equilibrium crystallisation and dissolution temperatures, van't Hoff fitting parameters, relative critical undercooling (u_c_) values as a function of q as well as the calculated values of K_G_ and α_det_.

**Specifications Table**

**Table:** 

Subject	Chemical Engineering
Specific subject area	Crystallisation and Fuels
Type of data	Tables
How the data were acquired	Data was acquired from polythermal crystallisation experiments using the Technobis Crystal16, computationally using a dedicated systematic search algorithm, and thermodynamic data was collected using a Mettler Toledo DSC 1 with the polythermal method.
Data format	Raw & Analysed
Description of data collection	In all Crystal16 experiments solutions were homogenised above the dissolution point with agitation before crystallisation with each polythermal cycle being repeated 4 times. 7 cooling rates from 0.25 - 5°C/min were used, with dissolution and crystallisation points being obtained from a Boltzmann fitting of the turbidity/temperature data with dissolution taken when %T reached 99% and crystallisation taken when %T reached 90%. In all DSC experiments the samples were also homogenised above their melting point before crystallisation with each polythermal cycle being repeated 3 times at 2°C/min.
Data source location	University of Leeds, Leeds, West Yorkshire, United Kingdom
Data accessibility	Data is available at the Research Data Leeds Repository https://doi.org/10.5518/1299
Related research article	A. S. M. Jackson. D. Goberdhan, P. J. Dowding, K. J. Roberts, Solution Crystallisation of Single and Mixed n-Alkanes, within the Homologous Series C_16_ to C_23_ from Representative Hydrocarbon Fuel Solvents, Fluid Phase Equilibria, 2022, 113705

## Value of the Data

• Crystallisation of multicomponent n-alkane waxes in diesel fuels under cold weather conditions is a current technological problem, where the influence of the multicomponent nature of the fuel on the crystallisation behavior and its relation to additive effectiveness is not well understood.
• Crystallisation of single n-alkanes, C_16_H_34_ – C_23_H_48_, and model multicomponent mixtures of these compounds in both toluene and dodecane, provides and insight into the crystallisation behavior of these compounds in representative diesel solvents.
• The data enables the nucleation mechanism to be determined as well as the calculation of key nucleation kinetic parameters, leading to a greater understanding of how crystallisation occurs in these systems and the impact of solute and solvent nature.
• Future work on a more complete understanding of how the compositional nature of these fuels affects the nucleation mechanism, its kinetics and its impact on additive effectiveness, can be built from this founding work.

## 1 Objective

The crystallisation of *n*-alkanes in diesel fuels is a common problem facing the petrochemical industry. The multicomponent nature of these fuels and its influence on crystallisation, provides a complex challenge for additive manufactures in designing additives that combat this behaviour. In order to develop improved additives, it is vital to understand crystallisation in these systems. A recent article published by the authors investigated the crystallisation of single *n*-alkanes, C_16_H_34_-C_23_H_48_ and model multicomponent mixtures of these, from representative fuel solvents, providing insight into the crystallisation behaviour of compounds representative of diesel. The information presented here expands upon the information in the referenced article, by providing a complete dataset of solubility and nucleation kinetic values for the *n*-alkanes C_16_H_34_-C_23_H_48_. This allows the published work to be used as a comprehensive reference for the crystallisation of these *n*-alkanes.

## 2 Data Description

### 2.1 Solubility data

Average crystallisation (Tc) and dissolution (Td) temperatures for each single alkane solute and a mixture of the 8 alkanes representative of diesel fuel, at 7 cooling rates (q), 0.25, 0.5, 1, 2, 3.2, 4 and 5°C/min, for each solution concentration are shown in Tables 1 and 2 for the solvents dodecane and toluene respectively. The temperature displayed is the average of 4 polythermal cycles, along with the calculated standard deviations. The data provided highlights the influence of solute composition, concentration and cooling rate upon the crystallisation and dissolution behaviour of single and multicomponent alkane solutions. The equilibrium values of crystallisation (Tc,l) and dissolution (Te) temperature for the single alkanes and an 8-alkane mixture, in both toluene and dodecane over the range of compositions used are shown in Table 3 along with their associated errors. Both were calculated from the intercept value of a linear fitting of the Tc or Td data with q. The data highlights solubility and MSZW of the alkanes and its dependence on solute composition, concentration and solvent. The parameters of the van't Hoff fitting of the equilibrium solubility data for the single alkanes and an 8-alkane mixture in toluene and dodecane and their associated errors are shown in Table 4, along with the predicted solubility temperature at 0.165xi according to the model. This data highlights the difference in solubility normalised to the same concentration for all system as well as the applicability of the van't Hoff model.

**Table 1:** Average crystallisation, T_c_, and dissolution, T_d_, temperatures with their associated errors from 4 repeats of a polythermal crystallisation and dissolution cycle for 8 single n-alkanes and a mixture of 8C_n_ in dodecane at 7 cooling rates. Errors are the standard deviation from 4 repeat measurements of T_c_ and T_d_ at each cooling rate and concentration.

	q	T_c_	*±T_c_*	T_d_	*±T_d_*	T_c_	*±T_c_*	T_d_	*±T_d_*	T_c_	*±T_c_*	T_d_	*±T_d_*	T_c_	*±T_c_*	T_d_	*±T_d_*	T_c_	*±T_c_*	T_d_	*±T_d_*
Solute	°C/min	°C	*±°C*	°C	*±°C*	°C	*±°C*	°C	*±°C*	°C	*±°C*	°C	*±°C*	°C	*±°C*	°C	*±°C*	°C	*±°C*	°C	*±°C*
C16		0.231 x_i_				0.260 x_i_				0.286 x_i_				0.311 x_i_				0.197 x_i_			
	0.25	-3.0	0.3	0.7	0.3	-1.5	0.3	2.2	0.1	0.5	0.2	3.5	0.3	0.9	0.5	4.5	0.2	-6.1	0.2	-1.2	0.1
	0.50	-3.5	0.2	1.2	0.1	-1.5	0.8	3.0	0.1	0.7	0.1	4.3	0.2	1.1	0.5	5.1	0.2	-5.7	0.5	-0.3	0.0
	1.00	-5.0	0.3	2.1	0.3	-2.5	0.6	4.2	0.5	-0.8	0.3	5.5	0.3	0.9	0.2	6.3	0.3	-7.4	0.3	1.5	0.2
	2.00	-5.4	0.4	7.2	0.2	-3.8	0.1	8.0	0.2	-2.2	0.3	9.2	0.5	-1.5	0.5	9.3	0.5	-8.1	0.1	5.3	0.2
	3.20	-5.8	0.4	8.3	0.5	-4.4	0.3	10.8	0.5	-1.9	0.2	11.5	0.5	-1.6	0.4	11.2	0.5	-8.3	0.4	8.8	0.8
	4.00	-4.5	0.3	8.2	0.4	-3.0	0.3	11.3	0.9	-1.7	0.2	11.4	0.4	-0.5	0.1	13.1	0.5	-9.5	0.1	10.8	0.3
	5.00	-6.3	0.2	14.6	1.0	-4.9	0.2	15.3	1.0	-3.9	0.1	19.3	1.4	-3.5	0.3	17.3	1.1	-9.3	0.3	13.3	0.3

C17		0.221 x_i_				0.249 x_i_				0.274 x_i_				0.298 x_i_				0.197 x_i_			
	0.25	-0.6	0.1	1.5	0.1	0.9	0.2	3.1	0.3	2.2	0.3	4.7	0.0	2.7	0.1	5.3	0.2	-1.8	0.1	0.1	0.1
	0.50	-1.0	0.2	2.1	0.2	0.9	0.2	3.7	0.1	2.3	0.3	5.3	0.2	3.0	0.2	6.1	0.2	-1.7	0.2	0.8	0.1
	1.00	-2.1	0.2	3.3	0.3	0.0	0.2	5.5	0.5	1.6	0.4	7.0	0.3	2.2	0.2	7.9	0.2	-2.6	0.1	3.5	0.3
	2.00	-2.5	0.1	9.1	0.6	-0.9	0.3	10.8	0.3	0.3	0.2	11.5	0.2	0.7	0.3	11.9	1.1	-3.3	0.1	7.9	0.6
	3.20	-3.4	0.2	10.6	2.8	-1.3	0.2	12.1	2.3	0.1	0.1	13.9	1.7	0.8	0.1	13.5	1.0	-3.8	0.1	12.2	0.3
	4.00	-2.6	0.2	8.1	0.4	-1.2	0.2	13.1	1.4	0.1	0.3	14.0	0.1	1.0	0.2	15.3	0.4	-4.9	0.2	14.5	0.2
	5.00	-3.9	0.2	20.6	0.7	-2.5	0.4	21.7	1.1	-1.8	0.2	20.7	1.2	-1.4	0.3	22.2	2.2	-4.9	0.3	18.1	0.3

C18		0.211 x_i_				0.238 x_i_				0.263 x_i_				0.287 x_i_				0.182 x_i_			
	0.25	7.9	0.3	10.8	0.1	9.9	0.4	12.4	0.1	10.9	0.2	13.5	0.0	11.7	0.4	14.6	0.3	6.1	0.2	8.7	0.1
	0.50	8.0	0.2	11.3	0.1	9.4	0.3	12.9	0.2	10.9	0.3	14.2	0.5	12.0	0.2	15.1	0.1	6.1	0.3	9.2	0.1
	1.00	6.7	0.4	12.8	0.4	8.9	0.4	14.7	0.2	10.4	0.2	15.8	0.3	11.2	0.1	17.0	0.4	5.3	0.4	10.2	0.1
	2.00	6.1	0.2	14.6	0.2	8.1	0.6	16.5	0.4	8.7	0.1	18.2	0.3	10.0	0.7	19.2	0.4	4.4	0.2	12.9	0.2
	3.20	5.7	0.4	18.1	0.4	7.5	0.4	19.4	0.1	8.9	0.4	20.5	0.4	9.9	0.2	21.9	0.4	3.8	0.2	16.7	0.4
	4.00	4.2	0.3	18.2	0.4	6.1	0.3	21.7	0.5	7.9	0.2	21.3	0.3	8.7	0.4	25.1	1.6	3.4	0.4	18.1	0.7
	5.00	3.7	0.1	23.1	0.2	5.4	0.5	25.0	0.4	5.9	0.3	26.1	0.7	6.9	0.3	27.7	0.6	2.5	0.7	21.1	0.7
C19		0.202 x_i_				0.229 x_i_				0.253 x_i_				0.276 x_i_				0.182 x_i_			
	0.25	10.1	0.3	12.0	0.2	11.4	0.2	13.5	0.1	12.7	0.2	14.7	0.1	13.7	0.1	15.7	0.4	8.3	0.1	10.3	0.2
	0.50	9.7	0.1	12.4	0.1	11.2	0.1	13.6	0.1	12.3	0.1	15.3	0.3	13.4	0.1	16.0	0.1	8.1	0.2	10.6	0.1
	1.00	8.7	0.4	13.9	0.6	10.6	0.3	15.6	0.5	11.9	0.3	16.9	0.3	13.0	0.1	18.0	0.2	7.7	0.1	11.6	0.2
	2.00	8.1	0.2	16.3	0.7	9.4	0.1	17.5	0.4	10.8	0.3	19.2	0.2	11.7	0.3	20.2	0.6	6.6	0.3	14.6	0.3
	3.20	7.8	0.1	18.7	0.3	9.1	0.1	20.5	0.5	10.4	0.1	21.9	0.3	11.7	0.1	22.1	0.3	6.3	0.3	16.8	0.3
	4.00	6.1	0.2	19.4	1.5	8.0	0.0	21.5	0.2	9.4	0.3	23.6	0.4	11.0	0.1	24.6	0.5	5.3	0.2	20.1	0.7
	5.00	6.1	0.2	24.0	0.6	7.0	0.2	25.3	0.2	8.2	0.3	26.9	0.6	8.9	0.2	27.1	1.0	4.5	0.2	22.9	0.6

C20		0.194 x_i_				0.220 x_i_				0.243 x_i_				0.266 x_i_				0.168 x_i_			
	0.25	17.3	0.4	19.4	0.2	18.5	0.3	20.6	0.2	19.4	0.2	21.8	0.3	20.7	0.1	22.6	0.3	14.0	1.4	17.4	0.3
	0.50	17.0	0.2	19.7	0.4	18.1	0.2	21.2	0.4	19.3	0.1	22.3	0.5	20.4	0.2	23.1	0.3	15.0	0.1	17.7	0.1
	1.00	16.5	0.4	21.5	0.5	17.7	0.4	22.5	0.1	19.0	0.1	23.7	0.3	20.0	0.3	24.8	0.2	14.5	0.1	18.3	0.3
	2.00	15.5	0.4	23.2	0.2	16.5	0.2	24.1	0.3	17.8	0.3	25.5	0.1	18.9	0.2	27.1	0.6	13.6	0.3	19.8	0.1
	3.20	14.9	0.2	24.0	0.4	16.6	0.2	25.1	0.5	17.4	0.2	27.3	0.1	18.6	0.1	28.1	0.1	13.0	0.2	21.8	0.5
	4.00	13.9	0.1	27.3	0.2	15.1	0.2	28.4	0.5	16.5	0.3	29.9	0.6	17.6	0.1	31.0	0.7	12.4	0.1	23.7	0.6
	5.00	12.8	0.2	29.2	0.2	14.1	0.2	30.2	0.6	15.4	0.2	31.4	0.4	16.0	0.1	33.2	0.7	11.8	0.1	25.6	0.3

C21		0.187 x_i_				0.211 x_i_				0.235 x_i_				0.256 x_i_				0.168 x_i_			
	0.25	18.8	0.1	20.7	0.0	20.1	0.1	22.1	0.2	21.1	0.2	23.2	0.1	22.1	0.1	24.0	0.2	16.4	0.1	18.5	0.2
	0.50	20.0	0.1	22.7	0.1	21.2	0.2	24.2	0.1	22.2	0.1	25.4	0.1	23.3	0.1	26.1	0.2	16.2	0.2	19.4	0.1
	1.00	17.8	0.3	23.0	0.8	19.6	0.2	23.9	0.4	20.6	0.3	25.9	0.7	21.5	0.3	26.1	0.1	15.8	0.3	19.8	0.0
	2.00	16.8	0.2	24.3	0.6	18.5	0.2	25.8	0.7	19.5	0.4	27.6	0.8	20.4	0.3	29.1	0.4	15.3	0.1	21.3	0.3
	3.20	16.3	0.2	26.1	0.4	17.9	0.0	27.7	0.6	19.0	0.1	28.9	0.6	20.1	0.2	30.4	0.3	14.4	0.2	24.4	0.3
	4.00	15.0	0.2	28.0	1.1	17.0	0.2	29.7	0.6	18.4	0.2	32.0	1.4	19.4	0.3	33.0	0.5	13.8	0.0	25.4	0.6
	5.00	14.5	0.1	30.1	0.6	16.2	0.2	31.4	0.6	16.9	0.1	33.9	0.6	17.8	0.2	35.5	0.6	13.5	0.2	27.1	1.2
C22		0.180 x_i_				0.204 x_i_				0.226 x_i_				0.248 x_i_				0.155 x_i_			
	0.25	24.5	0.2	26.5	0.3	25.7	0.0	27.6	0.3	26.6	0.1	28.6	0.2	27.9	0.2	29.6	0.3	21.4	0.3	23.4	0.1
	0.50	23.7	0.3	26.5	0.4	25.1	0.1	28.0	0.3	26.3	0.1	28.8	0.2	27.3	0.2	30.2	0.2	20.9	0.1	23.6	0.2
	1.00	23.4	0.2	28.3	0.1	24.9	0.2	29.6	0.3	26.0	0.2	30.8	0.1	27.4	0.2	32.5	0.7	20.9	0.1	24.5	0.1
	2.00	22.6	0.3	30.3	0.5	23.9	0.2	32.2	0.3	24.9	0.3	33.0	0.7	26.0	0.3	35.2	0.2	20.2	0.1	26.8	0.3
	3.20	21.9	0.3	32.3	0.5	23.4	0.1	33.6	0.3	24.8	0.1	34.3	0.4	25.7	0.2	35.9	0.4	19.1	0.1	28.6	0.2
	4.00	20.9	0.3	35.3	0.9	22.8	0.3	35.3	0.6	24.0	0.2	37.0	0.7	25.3	0.2	37.1	0.5	19.0	0.1	29.5	0.2
	5.00	20.5	0.2	35.9	0.8	21.7	0.2	37.7	0.5	22.7	0.0	38.5	0.2	23.8	0.3	38.7	0.1	18.3	0.0	31.9	0.4

C23		0.174 x_i_				0.197 x_i_				0.219 x_i_				0.240 x_i_				0.155 x_i_			
	0.25	26.0	0.2	28.0	0.1	27.4	0.1	29.2	0.0	28.3	0.2	30.1	0.1	29.2	0.2	31.1	0.1	23.3	0.2	25.3	0.1
	0.50	25.1	0.4	28.5	0.1	26.8	0.1	29.5	0.1	27.9	0.1	30.6	0.1	28.8	0.0	31.7	0.1	23.0	0.1	25.6	0.1
	1.00	25.2	0.3	29.8	0.1	26.6	0.2	30.7	0.1	27.7	0.2	32.2	0.4	28.7	0.3	33.4	0.5	22.7	0.2	26.7	0.0
	2.00	24.5	0.2	31.4	0.3	25.6	0.3	32.6	0.5	26.7	0.2	34.0	0.4	27.5	0.3	35.3	0.5	22.2	0.2	28.9	0.5
	3.20	23.2	0.2	32.9	0.4	24.9	0.2	34.2	0.8	26.1	0.1	34.9	0.8	26.8	0.4	36.8	0.7	21.3	0.4	29.6	0.4
	4.00	22.4	0.1	35.3	0.4	24.5	0.1	36.7	0.7	25.8	0.2	37.6	0.5	26.6	0.2	39.6	0.6	20.7	0.1	30.9	0.1
	5.00	22.1	0.2	35.8	0.6	23.3	0.2	37.7	0.6	24.3	0.2	39.0	0.1	24.8	0.2	40.7	0.6	20.2	0.1	33.0	0.3

8Cn		0.100 x_i_				0.200 x_i_				0.300 x_i_				0.496 x_i_							
	0.25	-4.1	0.2	-2.8	0.3	3.0	0.1	5.0	0.5	8.2	0.2	9.4	0.3	15.5	0.1	16.8	0.1				
	0.50	-4.1	0.1	-2.3	0.8	3.4	0.1	5.0	0.1	8.1	0.2	9.6	0.1	-	-	-	-				
	1.00	-5.2	0.2	-1.8	0.3	2.7	0.3	5.6	0.2	7.7	0.3	10.7	0.1	15.3	0.1	19.1	0.1				
	2.00	-5.9	0.4	2.7	1.3	2.1	0.3	7.1	0.1	6.5	0.3	12.1	0.3	14.5	0.1	21.3	0.3				
	3.20	-5.2	1.1	3.8	1.5	1.3	0.2	9.4	0.3	6.0	0.1	14.0	0.2	13.8	0.2	24.5	0.2				
	4.00	-5.2	0.3	3.6	2.3	0.4	0.3	10.3	0.2	5.5	0.1	16.0	0.4	13.1	0.2	26.6	0.4				
	5.00	-6.6	0.2	10.0	1.5	-0.1	0.3	12.6	0.7	4.0	0.4	18.0	0.2	12.7	0.2	28.6	0.8

**Table 2:** Average crystallisation, T_c_, and dissolution, T_d_, temperatures with their associated errors from 4 repeats of a polythermal crystallisation and dissolution cycle for 8 single n-alkanes and a mixture of 8C_n_ in toluene at 7 cooling rates. Errors are the standard deviation from 4 repeat measurements of T_c_ and T_d_ at each cooling rate and concentration.

	q	T_c_	*±T_c_*	T_d_	*±T_d_*	T_c_	*±T_c_*	T_d_	*±T_d_*	T_c_	*±T_c_*	T_d_	*±T_d_*	T_c_	*±T_c_*	T_d_	*±T_d_*	T_c_	*±T_c_*	T_d_	*±T_d_*
Solute	°C/min	°C	*±°C*	°C	*±°C*	°C	*±°C*	°C	*±°C*	°C	*±°C*	°C	*±°C*	°C	*±°C*	°C	*±°C*	°C	*±°C*	°C	*±°C*
C16		0.124 x_i_				0.142 x_i_				0.159 x_i_				0.175 x_i_				0.200 x_i_			
	0.25	-5.4	0.2	-3.6	0.3	-4.2	0.2	-2.4	0.0	-3.0	0.1	-1.2	0.2	-2.5	0.2	-0.5	0.3	-0.8	0.1	1.2	0.2
	0.50	-5.6	0.2	-3.2	0.3	-4.2	0.1	-1.8	0.1	-3.1	0.2	-0.5	0.1	-2.4	0.2	0.3	0.2	-0.7	0.1	2.1	0.1
	1.00	-6.7	0.2	-2.0	0.5	-4.9	0.2	-0.5	0.4	-3.5	0.2	1.5	0.4	-2.6	0.3	2.0	0.2	-1.7	0.1	3.3	0.4
	2.00	-6.8	0.3	1.2	0.2	-5.5	0.1	2.4	0.3	-4.8	0.2	3.6	0.3	-4.1	0.4	4.5	0.2	-2.2	0.3	6.2	0.1
	3.20	-6.8	0.4	2.7	0.3	-5.4	0.4	3.6	0.1	-4.5	0.3	6.8	1.4	-4.0	0.2	6.5	0.3	-2.2	0.3	8.6	0.7
	4.00	-6.5	0.5	4.6	0.5	-4.8	0.1	5.6	0.7	-3.5	0.1	8.2	0.6	-2.8	0.1	8.8	0.9	-3.5	0.2	9.7	0.2
	5.00	-7.1	0.1	8.3	1.9	-6.2	0.1	8.0	0.3	-5.5	0.2	10.3	0.7	-5.1	0.2	11.4	0.2	-3.7	0.2	12.4	0.4

C17		0.118 x_i_				0.135 x_i_				0.151 x_i_				0.167 x_i_				0.201 x_i_			
	0.25	-5.0	0.1	-3.2	0.3	-3.4	0.1	-1.2	0.4	-2.3	0.3	-0.6	0.4	-1.6	0.1	0.3	0.2	0.9	0.1	2.6	0.1
	0.50	-4.9	0.1	-2.1	0.3	-3.4	0.1	-1.0	0.3	-2.3	0.1	0.0	0.4	-1.8	0.1	0.9	0.4	0.8	0.1	3.3	0.0
	1.00	-5.5	0.1	-1.7	0.4	-4.1	0.2	0.3	0.2	-2.7	0.1	1.7	0.2	-1.8	0.2	2.6	0.2	0.5	0.3	4.7	0.3
	2.00	-5.8	0.3	1.4	0.6	-4.5	0.2	2.7	0.4	-3.9	0.3	3.9	0.9	-3.7	0.2	5.3	0.2	0.0	0.2	7.7	0.3
	3.20	-6.1	0.3	2.6	1.1	-4.5	0.1	3.9	0.9	-3.7	0.1	6.2	0.1	-3.3	0.3	8.0	0.4	-0.5	0.2	9.6	0.3
	4.00	-5.2	0.1	3.7	0.7	-4.2	0.1	6.0	0.7	-2.9	0.1	8.1	0.5	-2.1	0.1	9.2	0.9	-1.2	0.1	11.6	0.9
	5.00	-6.2	0.4	6.4	2.6	-5.3	0.1	8.8	1.0	-4.6	0.2	11.3	1.5	-4.7	0.2	13.3	1.3	-1.7	0.1	13.4	0.4

C18		0.112 x_i_				0.128 x_i_				0.144 x_i_				0.159 x_i_				0.185 x_i_			
	0.25	3.6	0.2	5.7	0.4	4.8	0.3	6.8	0.3	5.9	0.3	7.8	0.5	6.8	0.2	8.5	0.2	8.4	0.0	10.3	0.2
	0.50	3.2	0.3	6.3	0.5	4.7	0.1	7.1	0.2	5.8	0.1	8.4	0.5	6.5	0.2	9.0	0.2	8.3	0.1	10.9	0.1
	1.00	2.1	0.3	7.7	0.4	3.9	0.3	8.5	0.4	5.4	0.2	10.2	0.4	6.4	0.2	10.5	0.3	8.0	0.2	12.1	0.2
	2.00	2.2	0.3	9.9	1.1	3.3	0.3	11.0	0.5	4.2	0.3	11.5	0.4	4.8	0.5	9.8	6.9	7.3	0.1	14.6	0.3
	3.20	1.3	0.2	11.6	0.5	2.9	0.3	12.8	0.3	4.1	0.2	13.6	0.3	4.8	0.2	15.0	0.4	6.6	0.1	17.3	0.4
	4.00	1.3	0.1	13.1	0.6	2.3	0.2	15.8	0.9	3.8	0.2	17.1	0.7	4.5	0.1	17.7	0.1	6.2	0.1	18.5	0.2
	5.00	0.0	0.2	15.2	0.4	1.2	0.1	17.0	0.5	2.0	0.2	19.1	1.3	2.0	0.3	21.5	0.6	5.8	0.1	20.7	0.2
C19		0.107 x_i_				0.122 x_i_				0.137 x_i_				0.152 x_i_				0.185 x_i_			
	0.25	0.4	7.0	0.2	6.4	0.2	8.2	0.1	7.3	0.3	9.2	0.1	8.0	0.3	9.9	0.5	9.9	0.1	11.4	0.1	0.4
	0.50	0.1	7.4	0.4	6.0	0.1	8.7	0.2	7.1	0.1	9.5	0.3	7.8	0.2	10.3	0.3	9.4	0.1	12.3	0.2	0.1
	1.00	0.3	9.1	0.4	5.6	0.4	10.1	0.5	6.9	0.2	11.2	0.2	7.5	0.4	12.8	0.2	9.3	0.2	14.3	0.4	0.3
	2.00	0.1	12.0	1.0	4.7	0.2	12.9	1.0	5.5	0.2	13.8	0.4	6.2	0.2	14.6	0.3	8.6	0.3	16.7	0.3	0.1
	3.20	0.2	13.1	1.2	4.0	0.3	14.5	0.4	5.6	0.2	15.3	0.2	6.0	0.1	16.8	1.2	7.6	0.2	19.9	0.2	0.2
	4.00	0.2	15.0	0.2	3.9	0.1	17.1	1.2	5.2	0.2	19.1	1.2	5.6	0.1	19.8	0.7	7.1	0.1	21.3	0.3	0.2
	5.00	0.4	16.7	0.8	2.6	0.1	17.8	0.7	3.4	0.3	20.4	0.8	3.7	0.3	22.1	1.3	6.8	0.2	22.7	0.4	0.4

C20		0.102 x_i_				0.117 x_i_				0.131 x_i_				0.145 x_i_				0.171 x_i_			
	0.25	11.5	0.3	13.7	0.1	12.8	0.4	14.8	0.1	14.0	0.1	15.8	0.1	14.7	0.2	16.7	0.1	15.9	0.1	17.6	0.2
	0.50	11.3	0.1	14.1	0.0	12.6	0.1	15.4	0.2	13.5	0.1	16.3	0.2	14.5	0.1	17.7	0.4	15.8	0.1	18.3	0.1
	1.00	10.6	0.2	16.2	0.6	12.3	0.2	17.8	0.3	13.5	0.2	18.2	0.1	14.3	0.1	19.7	0.3	15.3	0.2	20.6	0.4
	2.00	10.1	0.2	18.2	0.9	11.3	0.5	19.3	0.1	12.4	0.3	22.1	1.0	12.9	0.3	22.5	0.3	14.8	0.1	22.6	0.1
	3.20	9.4	0.0	20.9	1.8	10.7	0.1	21.4	0.2	11.9	0.3	22.9	0.7	12.8	0.2	24.2	0.1	14.2	0.0	24.4	0.2
	4.00	8.2	0.1	23.9	1.2	9.5	0.2	24.3	1.1	11.6	0.3	24.7	0.2	12.0	0.2	26.8	0.0	13.3	0.3	27.0	0.6
	5.00	7.9	0.2	25.2	1.8	9.0	0.1	26.0	0.3	9.9	0.2	27.7	0.3	10.2	0.1	28.9	0.3	13.0	0.1	28.4	0.2

C21		0.098 x_i_				0.112 x_i_				0.126 x_i_				0.140 x_i_				0.171 x_i_			
	0.25	13.1	0.3	15.2	0.5	14.3	0.2	16.0	0.3	14.9	0.2	16.9	0.5	15.9	0.1	18.1	0.4	17.7	0.2	19.1	0.5
	0.50	12.8	0.1	15.2	0.6	13.9	0.1	16.1	0.2	14.9	0.1	17.6	0.4	15.7	0.1	18.2	0.9	17.4	0.2	19.5	0.4
	1.00	11.9	0.4	16.2	0.2	13.6	0.5	17.9	0.6	14.8	0.2	19.4	0.6	15.6	0.3	20.2	0.9	16.9	0.2	21.2	0.1
	2.00	11.2	0.2	18.6	0.3	12.7	0.2	20.9	0.8	13.6	0.3	21.7	1.2	14.4	0.4	22.9	0.8	16.2	0.1	24.1	0.3
	3.20	10.6	0.1	20.7	0.2	12.1	0.3	23.4	0.4	13.1	0.2	23.9	1.1	13.9	0.2	24.5	0.1	15.3	0.1	26.8	0.6
	4.00	9.3	0.2	22.8	1.0	10.6	0.2	24.9	0.7	11.2	0.1	26.1	0.4	12.4	0.3	27.0	0.8	14.8	0.1	28.1	0.3
	5.00	9.2	0.0	26.0	0.5	10.0	0.2	27.3	1.0	11.0	0.1	28.3	0.6	11.8	0.2	29.2	0.3	14.4	0.3	30.4	0.3
C22		0.094 x_i_				0.107 x_i_				0.121 x_i_				0.134 x_i_				0.158 x_i_			
	0.25	18.2	0.3	20.4	0.3	19.4	0.1	21.5	0.3	20.3	0.2	22.5	0.2	21.6	0.2	23.5	0.2	22.3	0.1	24.0	0.1
	0.50	17.7	0.2	21.0	0.2	19.0	0.2	21.9	0.4	20.1	0.1	22.9	0.1	21.0	0.2	24.1	0.1	21.9	0.2	24.5	0.1
	1.00	17.7	0.4	22.3	0.2	18.9	0.2	23.6	0.2	20.0	0.2	25.3	0.6	21.2	0.3	26.1	0.2	21.8	0.1	26.1	0.2
	2.00	16.5	0.2	25.1	0.8	18.0	0.4	26.4	0.3	18.8	0.3	27.3	0.5	19.8	0.5	29.7	1.2	21.1	0.1	28.4	0.3
	3.20	15.8	0.2	27.1	0.2	17.3	0.1	27.9	0.4	18.4	0.2	29.0	0.4	19.5	0.2	30.8	0.4	20.4	0.1	30.5	0.2
	4.00	14.9	0.4	28.4	0.1	16.8	0.3	29.7	0.6	18.1	0.3	31.3	0.3	19.0	0.1	32.9	0.3	20.2	0.1	32.3	0.2
	5.00	14.4	0.1	30.3	0.1	15.9	0.3	32.7	0.4	16.8	0.3	33.8	0.4	17.4	0.2	35.9	0.3	19.4	0.2	34.1	0.2

C23		0.090 x_i_				0.103 x_i_				0.116 x_i_				0.129 x_i_				0.158 x_i_			
	0.25	19.5	0.2	21.9	0.5	20.9	0.3	22.7	0.3	21.6	0.2	23.6	0.2	22.7	0.1	24.5	0.6	23.7	0.0	25.4	0.1
	0.50	19.2	0.2	22.1	0.2	20.4	0.3	22.6	0.4	21.5	0.3	24.5	0.5	22.4	0.2	25.4	0.7	23.5	0.3	25.8	0.5
	1.00	18.9	0.3	23.4	0.3	20.5	0.3	25.1	0.5	21.4	0.1	25.7	0.5	22.6	0.3	27.2	0.5	23.3	0.2	27.7	0.9
	2.00	18.1	0.3	26.8	0.7	19.5	0.2	26.8	0.6	20.6	0.2	28.9	1.1	21.3	0.3	30.5	0.7	22.7	0.1	30.0	0.9
	3.20	17.5	0.2	28.4	1.4	18.8	0.1	29.5	1.3	19.6	0.2	29.8	0.1	20.9	0.2	31.3	1.4	21.6	0.3	32.2	0.1
	4.00	16.3	0.5	30.2	1.4	18.1	0.3	33.5	0.3	19.4	0.2	33.7	1.9	20.6	0.1	32.7	0.3	21.5	0.1	33.5	0.2
	5.00	15.9	0.3	32.9	2.3	17.4	0.3	32.9	0.3	18.3	0.2	34.6	1.8	18.5	0.3	35.5	0.4	20.8	0.2	35.2	0.3

8Cn		0.100 x_i_				0.199 x_i_				0.297 x_i_				0.500 x_i_							
	0.25	-1.2	0.1	1.6	0.1	4.4	0.1	6.1	0.1	8.6	0.1	9.6	0.5	15.4	0.1	16.5	0.1				
	0.50	-1.3	0.0	2.1	0.1	4.2	0.1	6.2	0.0	8.4	0.0	10.2	0.0	15.1	0.1	17.4	0.1				
	1.00	-2.1	0.1	2.2	0.1	3.8	0.1	6.8	0.1	8.1	0.1	11.2	0.1	14.9	0.1	18.4	0.1				
	2.00	-2.3	0.1	3.4	0.2	3.4	0.1	8.1	0.1	7.5	0.1	13.5	0.1	14.1	0.1	21.4	0.1				
	3.20	-3.1	0.1	4.5	0.2	2.8	0.0	10.1	0.1	6.9	0.1	15.0	0.2	13.3	0.1	24.0	0.3				
	4.00	-4.0	0.1	5.4	0.4	2.0	0.1	11.2	0.2	6.3	0.1	17.7	0.9	13.0	0.1	25.3	0.1				
	5.00	-3.8	0.2	6.3	0.2	1.7	0.0	12.4	0.2	5.8	0.3	19.2	0.5	12.2	0.1	28.0	0.5

**Table 3:** Equilibrium values of crystallisation, T_c,l_, and dissolution, T_e_, temperature for 8 single alkanes and a mixture of 8C_n_ in toluene and dodecane determined for each concentration from the extrapolation to the 0°c/min cooling rate linear fitting of T_c_ and T_d_ with cooling rate. Errors in T_c,l_ and T_e_ are the error in the intercept of these linear fits.

	*Toluene*					*Dodecane*				
Solute	Conc	T_c,l_	*±T_c,l_*	T_e_	*±T_e_*	Conc	T_c,l_	*±T_c,l_*	T_e_	*±T_e_*
	x_i_	°C	*±°C*	°C	*±°C*	x_i_	°C	*±°C*	°C	*±°C*
C16	0.124	-5.80	0.29	-4.30	0.37	0.231	-3.65	0.54	0.03	0.97
	0.142	-4.30	0.30	-2.70	0.30	0.260	-1.68	0.48	1.77	0.45
	0.159	-3.12	0.46	-1.45	0.26	0.286	0.48	0.50	2.63	1.02
	0.175	-2.36	0.48	-0.81	0.23	0.311	1.24	0.59	3.74	0.41
	0.200	-0.72	0.23	0.99	0.27	0.197	-6.07	0.36	-1.60	0.31

C17	0.118	-5.07	0.26	-3.31	0.34	0.221	-0.92	0.34	0.41	1.98
	0.135	-3.48	0.23	-1.82	0.29	0.249	0.90	0.25	2.14	1.18
	0.151	-2.30	0.38	-1.07	0.22	0.274	2.41	0.32	4.01	0.85
	0.167	-1.61	0.56	-0.24	0.33	0.298	3.01	0.43	4.52	0.87
	0.201	1.09	0.06	2.35	0.27	0.197	-1.70	0.18	-0.54	0.35

C18	0.112	3.43	0.25	5.47	0.19	0.211	8.05	0.25	10.11	0.54
	0.128	4.89	0.14	6.22	0.28	0.238	9.99	0.17	11.69	0.27
	0.144	6.10	0.28	7.21	0.40	0.263	11.28	0.34	13.03	0.51
	0.159	7.03	0.39	7.13	0.89	0.287	12.22	0.29	13.86	0.26
	0.185	8.52	0.06	9.91	0.17	0.182	6.23	0.12	7.81	0.20

C19	0.107	4.85	0.17	6.87	0.38	0.202	9.98	0.27	11.33	0.48
	0.122	6.37	0.15	7.94	0.39	0.229	11.56	0.17	12.76	0.32
	0.137	7.47	0.29	8.56	0.41	0.253	12.81	0.16	14.13	0.19
	0.152	8.23	0.28	9.45	0.36	0.276	13.93	0.33	15.18	0.24
	0.185	9.87	0.11	11.44	0.42	0.182	8.45	0.13	9.19	0.33

C20	0.102	11.65	0.14	13.21	0.32	0.194	17.48	0.12	18.89	0.44
	0.117	13.01	0.11	14.62	0.36	0.220	18.59	0.24	20.17	0.42
	0.131	14.10	0.22	15.62	0.55	0.243	19.68	0.14	21.39	0.22
	0.145	15.01	0.27	16.69	0.37	0.266	20.93	0.22	22.23	0.36
	0.171	16.03	0.09	17.59	0.37	0.168	14.85	0.25	16.66	0.20

C21	0.098	13.06	0.21	14.14	0.30	0.187	19.38	0.40	20.97	0.37
	0.112	14.51	0.18	15.45	0.27	0.211	20.74	0.31	22.30	0.37
	0.126	15.39	0.26	16.65	0.21	0.235	21.81	0.35	23.49	0.49
	0.140	16.29	0.18	17.55	0.30	0.256	22.81	0.3 9	24.09	0.43
	0.171	17.73	0.10	18.70	0.27	0.168	16.50	0.09	18.14	0.21

C22	0.094	18.28	0.10	20.19	0.27	0.180	24.34	0.15	25.92	0.35
	0.107	19.50	0.08	21.09	0.31	0.204	25.65	0.12	27.31	0.28
	0.121	20.50	0.16	22.24	0.35	0.226	26.75	0.20	28.21	0.33
	0.134	21.72	0.23	23.32	0.49	0.248	27.93	0.23	29.95	0.58
	0.158	22.32	0.09	23.70	0.20	0.155	21.43	0.12	22.89	0.19

C23	0.090	19.66	0.12	21.29	0.30	0.174	25.96	0.19	27.82	0.29
	0.103	21.04	0.11	22.08	0.66	0.197	27.40	0.14	28.73	0.23
	0.116	21.91	0.12	23.36	0.49	0.219	28.39	0.17	29.89	0.34
	0.129	23.03	0.31	24.67	0.53	0.129	29.36	0.22	30.91	0.33
	0.158	23.82	0.08	25.21	0.30	0.158	23.37	0.04	25.04	0.27

8Cn	0.100	-1.19	0.19	1.43	0.08	0.100	-4.29	0.37	-3.54	0.95
	0.199	4.51	0.08	5.53	0.11	0.200	3.46	0.12	4.16	0.21
	0.297	8.73	0.04	9.16	0.24	0.300	8.48	0.19	8.74	0.19
	0.500	15.47	0.06	16.16	0.21	0.496	15.78	0.10	16.41	0.17

**Table 4:** Fitting parameters of the van't Hoff modelled solubility to the equilibrium solubility determined for the 8 single and mixed n-alkanes in toluene and dodecane. Calculated solubility temperature from the van't Hoff parameters at 0.165x_i_ for 8 single alkanes and an 8C_n_ mixture in toluene and dodecane.

Solute	m	*±m*	c	*±c*	r^2^	Te @ 0.165x_i_ (°C)
Toluene						

16	-6851	337	23.39	1.24	0.99	-1.19
17	-7141	560	24.33	2.06	0.98	0.06
18	-8590	1761	28.70	6.28	0.89	8.47
19	-9668	602	32.31	2.13	0.99	10.31
20	-9593	666	31.20	2.31	0.99	17.54
21	-10028	806	32.56	2.78	0.98	18.70
22	-11625	1441	37.26	4.88	0.96	24.46
23	-11064	1558	35.18	5.25	0.94	26.06
8Cn	-8443	1207	28.58	4.29	0.96	4.75

Dodecane						

16	-6357	247	21.79	0.90	1.00	-3.72
17	-5654	465	19.14	1.69	0.98	-3.11
18	-5979	259	19.57	0.91	0.99	6.67
19	-5727	358	18.56	1.25	0.99	8.10
20	-7074	372	22.61	1.27	0.99	16.66
21	-6024	888	18.86	3.01	0.94	18.41
22	-6191	553	19.03	1.84	0.98	24.08
23	-6874	915	21.15	3.03	0.95	26.31

8Cn	-6308	275	21.12	0.99	1.00	2.02

### 2.2 Intermolecular interaction modelling data

Tables 5 and 6 show the total number of intermolecular interactions, found by the systematic search routine to have energy greater than the threshold value, -0.5, -1.0, -1.5, -2.0, -2.5 kCal, between a target and probe molecule for each of the alkane solutes with dodecane and toluene respectively. Tables 7 and 8 show the total energy of these interactions presented in Tables 5 and 6, for each alkane solute with dodecane and toluene respectively. Tables 9 and 10 show the strength of the largest energy interaction in kCal/mole of each alkane solute with dodecane and toluene respectively. This data highlights the relative interaction strength of each alkane molecule with the solvent and the impact of solute/solvent composition.

**Table 5:** Total number of interactions found on a 20-20-30 XYZ 7-11-8 UVW grid above a threshold energy value of -0.5, -1.0, -1.5, -2.0 and -2.5 kCal/mol between a target alkane molecule and dodecane solvent probe.

	# Interactions above threshold energy value				
Target	-0.5	-1.0	-1.5	-2.0	-2.5
C12	60404	14064	3624	1080	264
C16	76376	21014	5688	1840	624
C17	82094	22920	6348	1904	608
C18	89203	23895	6760	2264	688
C19	90255	26197	7304	2224	776
C20	91284	27588	7742	2496	864
C21	97093	29536	8224	2552	936
C22	102281	30936	8776	2952	1048
C23	107220	32924	9452	3024	1032

**Table 6:** Total number of interactions found on a 20-20-30 XYZ 7-11-8 UVW grid above a threshold energy value of -0.5, -1.0, -1.5, -2.0 and -2.5 kCal/mol between a target alkane molecule and toluene solvent probe.

	# Interactions above threshold energy value				
Target	-0.5	-1.0	-1.5	-2.0	-2.5
Toluene	3362	100	0	0	0
C16	11683	968	252	0	0
C17	11328	896	308	0	0
C18	12380	1024	324	0	0
C19	12930	1227	444	0	0
C20	13435	1360	424	0	0
C21	14722	1332	500	0	0
C22	15940	1396	468	0	0
C23	15691	1344	528	0	0

**Table 7:** Total energy of all interactions found above a threshold energy value (kCal/mol) between a target n-alkane molecule and a dodecane solvent probe using a 20-20-30 XYZ 7-11-8 UVW grid.

	Total Energy of Interactions above threshold energy value (kCal/mol)				
Target	-0.5	-1.0	-1.5	-2.0	-2.5
C12	-2684.863	-1646.261	-756.608	-307.454	-80.566
C16	-3732.212	-2558.771	-1343.897	-648.838	-265.534
C17	-3624.321	-2448.224	-1272.862	-565.566	-219.195
C18	-4045.527	-2855.254	-1548.536	-777.326	-295.728
C19	-3997.656	-2762.433	-1471.564	-668.505	-284.141
C20	-4101.573	-2877.946	-1598.704	-761.985	-326.3
C21	-4343.948	-3057.563	-1668.692	-792.146	-344.703
C22	-4471.435	-3231.902	-1847.019	-942.524	-393.577
C23	-4668.745	-3371.295	-1958.724	-933.428	-393.524

**Table 8:** Total energy of all interactions found above a threshold energy value (kCal/mol) between a target n-alkane molecule and a toluene solvent probe using a 30-30-30 XYZ 8-11-8 UVW grid.

	Total Energy of Interactions above threshold energy value (kCal/mol)				
Target	-0.5	-1.0	-1.5	-2.0	-2.5
Toluene	-452.172	-26.326	0	0	0
C16	-945.22	-341.371	-91.74	0	0
C17	-850.718	-262.136	-106.69	0	0
C18	-1055.033	-332.034	-96.476	0	0
C19	-996.742	-331.632	-126.257	0	0
C20	-979.949	-343.381	-113.676	0	0
C21	-1028.7	-380.623	-155.862	0	0
C22	-1150.68	-447.176	-154.001	0	0
C23	-1074.945	-381.449	-157.514	0	0

**Table 9:** Strength of the largest energy interaction found between a target n-alkane molecule and a dodecane solvent probe using a 20-20-30 XYZ 7-11-8 UVW grid.

Largest Energy Interaction	
Target	(kCal/mol)
C12	-3.495
C16	-3.877
C17	-3.836
C18	-3.94
C19	-3.867
C20	-3.957
C21	-3.888
C22	-3.973
C23	-3.889

**Table 10:** Strength of the largest energy interaction found between a target n-alkane molecule and a toluene solvent probe using a 30-30-30 XYZ 8-11-8 UVW grid.

Largest Energy Interaction	
Target	(kCal/mol)
Toluene	-1.108
C16	-1.75
C17	-1.678
C18	-1.754
C19	-1.686
C20	-1.755
C21	-1.691
C22	-1.758
C23	-1.689

### 2.3 Nucleation kinetics data

The natural log of the cooling rate (K/sec) and the natural log of the relative critical undercooling (u_c_) for each concentration for each alkane solute and an 8-alkane mixture, are shown in Tables 11 and 12 in dodecane and toluene respectively. The linear fitting of this data determines the nucleation mechanism according to the rule of 3 from the KBHR approach. The calculated values of the detectable fraction of crystallised volume (α_det_) and the growth rate constant (K_G_) for each concentration of alkane and 8-alkane mixture in dodecane and toluene are shown in Tables 13 and 14 respectively. This data is used in the calculation of the nucleation kinetic parameters for the instantaneous case according to the KBHR approach.

**Table 11:** Values of natural log of critical undercooling (lnu_c_) calculated at each cooling rate (lnq) for each concentration of 8 single alkanes and an 8C_n_ mixture in toluene according to the KBHR approach.

		ln*u_c_*				
	ln*q*	Conc A	Conc B	Conc C	Conc D	Conc E
C16		0.124	0.142	0.159	0.175	0.200
	-5.48	-5.472	-5.197	-5.144	-5.088	-5.031
	-4.79	-5.362	-5.227	-5.128	-5.165	-5.061
	-4.09	-4.718	-4.810	-4.898	-5.051	-4.608
	-3.40	-4.688	-4.576	-4.384	-4.424	-4.468
	-2.93	-4.684	-4.608	-4.486	-4.435	-4.453
	-2.71	-4.783	-4.846	-4.894	-4.927	-4.104
	-2.48	-4.571	-4.354	-4.206	-4.155	-4.058

C17		0.118	0.135	0.151	0.167	0.201
	-5.48	-5.102	-5.153	-5.414	-5.277	-5.271
	-4.79	-5.122	-5.142	-5.406	-5.190	-5.196
	-4.09	-4.812	-4.773	-5.140	-5.159	-4.994
	-3.40	-4.704	-4.616	-4.582	-4.373	-4.754
	-2.93	-4.557	-4.618	-4.654	-4.479	-4.569
	-2.71	-4.953	-4.735	-5.016	-4.988	-4.347
	-2.48	-4.550	-4.362	-4.333	-4.104	-4.224

C18		0.112	0.128	0.144	0.159	0.185
	-5.48	-5.023	-5.275	-5.337	-6.632	-5.258
	-4.79	-4.818	-5.203	-5.314	-6.108	-5.149
	-4.09	-4.421	-4.807	-5.027	-5.926	-4.973
	-3.40	-4.441	-4.571	-4.526	-4.773	-4.671
	-2.93	-4.210	-4.428	-4.488	-4.792	-4.457
	-2.71	-4.194	-4.275	-4.408	-4.672	-4.321
	-2.48	-3.932	-4.023	-3.975	-4.008	-4.224

C19		0.107	0.122	0.137	0.152	0.185
	-5.48	-4.986	-5.186	-5.383	-5.257	-5.196
	-4.79	-4.780	-4.979	-5.259	-5.137	-4.950
	-4.09	-4.472	-4.769	-5.120	-4.998	-4.876
	-3.40	-4.458	-4.453	-4.535	-4.474	-4.596
	-2.93	-4.180	-4.262	-4.556	-4.410	-4.314
	-2.71	-4.100	-4.232	-4.423	-4.307	-4.173
	-2.48	-3.913	-3.960	-3.995	-3.892	-4.119

C20		0.102	0.117	0.131	0.145	0.171
	-5.48	-5.133	-5.069	-5.177	-4.977	-5.120
	-4.79	-5.026	-4.949	-4.918	-4.875	-5.095
	-4.09	-4.712	-4.812	-4.899	-4.811	-4.842
	-3.40	-4.530	-4.468	-4.483	-4.348	-4.628
	-2.93	-4.325	-4.293	-4.356	-4.318	-4.453
	-2.71	-4.046	-4.040	-4.265	-4.115	-4.224
	-2.48	-3.983	-3.945	-3.921	-3.797	-4.140

C21		0.098	0.112	0.126	0.140	0.171
	-5.48	-5.641	-5.507	-5.121	-5.198	-5.723
	-4.79	-5.351	-5.250	-5.086	-5.083	-5.434
	-4.09	-4.844	-5.076	-5.029	-5.000	-5.093
	-3.40	-4.586	-4.643	-4.550	-4.526	-4.758
	-2.93	-4.387	-4.471	-4.415	-4.370	-4.461
	-2.71	-4.079	-4.082	-3.976	-4.025	-4.312
	-2.48	-4.056	-3.969	-3.932	-3.924	-4.213
C22		0.094	0.107	0.121	0.134	0.158
	-5.48	-4.971	-5.168	-5.020	-5.170	-5.369
	-4.79	-4.787	-4.940	-4.930	-4.873	-5.077
	-4.09	-4.752	-4.903	-4.885	-4.923	-5.056
	-3.40	-4.384	-4.555	-4.457	-4.445	-4.739
	-2.93	-4.204	-4.345	-4.351	-4.351	-4.503
	-2.71	-4.021	-4.224	-4.267	-4.237	-4.446
	-2.48	-3.928	-4.038	-3.996	-3.916	-4.229

C23		0.090	0.103	0.116	0.129	0.158
	-5.48	-5.117	-5.539	-5.130	-5.002	-5.282
	-4.79	-4.948	-5.176	-5.070	-4.870	-5.133
	-4.09	-4.810	-5.255	-5.025	-4.948	-5.038
	-3.40	-4.524	-4.741	-4.686	-4.493	-4.779
	-2.93	-4.348	-4.495	-4.369	-4.370	-4.424
	-2.71	-4.075	-4.317	-4.323	-4.290	-4.374
	-2.48	-4.002	-4.135	-4.069	-3.877	-4.222

8C_n_		0.100	0.199	0.297	0.500	
	-5.48	-4.644	-5.515	-6.286	-5.924	
	-4.79	-4.608	-5.376	-5.991	-5.608	
	-4.09	-4.363	-5.077	-5.582	-5.415	
	-3.40	-4.294	-4.864	-5.113	-4.951	
	-2.93	-4.112	-4.625	-4.820	-4.604	
	-2.71	-3.922	-4.371	-4.585	-4.521	
	-2.48	-3.957	-4.277	-4.422	-4.294

**Table 12:** Values of natural log of critical undercooling (lnu_c_) calculated at each cooling rate (lnq) for each concentration of 8 single alkanes and an 8C_n_ mixture in dodecane according to the KBHR approach.

	ln*q*	ln*u_c_*				
		Conc A	Conc B	Conc C	Conc D	Conc E
C16		0.231	0.260	0.286	0.311	0.197
	-5.48	-4.507	-4.419	-4.884	-4.572	-4.095
	-4.79	-4.339	-4.422	-4.952	-4.648	-4.186
	-4.09	-3.998	-4.169	-4.396	-4.580	-3.853
	-3.40	-3.909	-3.902	-4.043	-3.972	-3.738
	-2.93	-3.853	-3.800	-4.102	-3.950	-3.709
	-2.71	-4.097	-4.048	-4.157	-4.180	-3.535
	-2.48	-3.760	-3.717	-3.737	-3.646	-3.564

C17		0.221	0.249	0.274	0.298	0.197
	-5.48	-5.600	-5.405	-5.040	-5.047	-5.342
	-4.79	-5.303	-5.420	-5.104	-5.203	-5.418
	-4.09	-4.710	-4.847	-4.729	-4.794	-4.884
	-3.40	-4.555	-4.505	-4.302	-4.291	-4.576
	-2.93	-4.279	-4.370	-4.272	-4.305	-4.416
	-2.71	-4.497	-4.414	-4.268	-4.378	-4.145
	-2.48	-4.145	-4.093	-3.867	-3.845	-4.125

C18		0.211	0.238	0.263	0.287	0.182
	-5.48	-4.861	-5.082	-4.903	-4.900	-5.126
	-4.79	-4.902	-4.828	-4.880	-5.029	-5.075
	-4.09	-4.411	-4.626	-4.709	-4.673	-4.729
	-3.40	-4.248	-4.370	-4.198	-4.302	-4.420
	-2.93	-4.162	-4.226	-4.233	-4.272	-4.258
	-2.71	-3.878	-3.922	-4.018	-4.013	-4.145
	-2.48	-3.792	-3.806	-3.686	-3.721	-3.967
C19		0.202	0.229	0.253	0.276	0.182
	-5.48	-5.437	-5.319	-5.305	-5.280	-5.760
	-4.79	-5.167	-5.233	-5.050	-5.108	-5.594
	-4.09	-4.674	-4.887	-4.839	-4.905	-5.217
	-3.40	-4.492	-4.443	-4.462	-4.420	-4.677
	-2.93	-4.387	-4.363	-4.349	-4.404	-4.577
	-2.71	-4.006	-4.099	-4.111	-4.244	-4.293
	-2.48	-3.993	-3.901	-3.878	-3.820	-4.106

C20		0.194	0.220	0.243	0.266	0.168
	-5.48	-5.210	-5.145	-5.008	-5.239	-4.696
	-4.79	-5.045	-4.934	-4.934	-5.071	-5.183
	-4.09	-4.813	-4.790	-4.794	-4.902	-4.910
	-3.40	-4.448	-4.383	-4.403	-4.498	-4.556
	-2.93	-4.293	-4.400	-4.307	-4.386	-4.370
	-2.71	-4.061	-4.062	-4.088	-4.163	-4.217
	-2.48	-3.866	-3.877	-3.901	-3.854	-4.083

C21		0.187	0.211	0.235	0.256	0.168
	-5.48	-4.914	-4.882	-4.812	-4.991	-5.100
	-4.79	-5.665	-5.551	-5.467	-5.933	-5.039
	-4.09	-4.531	-4.692	-4.641	-4.757	-4.833
	-3.40	-4.262	-4.360	-4.314	-4.402	-4.645
	-2.93	-4.140	-4.211	-4.198	-4.313	-4.347
	-2.71	-3.900	-4.015	-4.075	-4.157	-4.206
	-2.48	-3.813	-3.882	-3.811	-3.859	-4.151

C22		0.180	0.204	0.226	0.248	0.155
	-5.48	-5.341	-5.226	-5.243	-4.996	-5.291
	-4.79	-4.909	-4.908	-5.071	-4.730	-5.018
	-4.09	-4.791	-4.816	-4.927	-4.768	-5.026
	-3.40	-4.514	-4.489	-4.506	-4.346	-4.690
	-2.93	-4.309	-4.339	-4.472	-4.268	-4.353
	-2.71	-4.089	-4.194	-4.281	-4.185	-4.339
	-2.48	-4.006	-3.988	-3.994	-3.894	-4.165

C23		0.174	0.197	0.219	0.240	0.155
	-5.48	-5.134	-5.456	-5.252	-5.178	-5.117
	-4.79	-4.717	-5.072	-5.006	-4.953	-4.975
	-4.09	-4.762	-4.955	-4.938	-4.931	-4.845
	-3.40	-4.508	-4.582	-4.542	-4.483	-4.636
	-2.93	-4.172	-4.369	-4.383	-4.305	-4.386
	-2.71	-4.017	-4.279	-4.310	-4.257	-4.235
	-2.48	-3.959	-4.019	-4.001	-3.913	-4.121

8C_n_		0.100	0.200	0.300	0.496	
	-5.48	-6.233	-5.478	-6.234	-5.775	
	-4.79	-6.182	-5.918	-6.141	-	
	-4.09	-5.081	-5.247	-5.653	-5.541	
	-3.40	-4.757	-4.885	-4.817	-5.040	
	-2.93	-5.074	-4.579	-4.641	-4.704	
	-2.71	-5.059	-4.301	-4.464	-4.464	
	-2.48	-4.477	-4.182	-4.078	-4.357

**Table 13:** Growth rate constant, K_G_ (m/s), and detectable fraction of crystallised volume, α_det_, calculated for each concentration of 8 alkanes and a mixture of 8 alkanes in dodecane for the KBHR approach instantaneous nucleation case.

Alkanes in Dodecane								
C_16_			C_17_			C_18_		
x_i_	K_G_	α_det_	x_i_	K_G_	α_det_	x_i_	K_G_	α_det_
0.231	5.05E-15	1.89E-04	0.221	4.76E-15	1.72E-04	0.211	4.66E-15	1.57E-04
0.260	5.93E-15	2.14E-04	0.249	5.59E-15	1.96E-04	0.238	5.46E-15	1.78E-04
0.286	6.79E-15	2.37E-04	0.274	6.43E-15	2.17E-04	0.263	6.27E-15	1.99E-04
0.311	7.67E-15	2.59E-04	0.298	7.25E-15	2.38E-04	0.287	7.08E-15	2.18E-04
0.197	4.09E-15	1.59E-04	0.197	4.11E-15	1.53E-04	0.182	3.84E-15	1.34E-04

C_19_			C_20_			C_21_		
x_i_	K_G_	α_det_	x_i_	K_G_	α_det_	x_i_	K_G_	α_det_

0.202	4.43E-15	1.44E-04	0.194	4.32E-15	1.33E-04	0.187	4.15E-15	1.22E-04
0.229	5.20E-15	1.65E-04	0.220	5.07E-15	1.52E-04	0.211	4.86E-15	1.40E-04
0.253	5.97E-15	1.84E-04	0.243	5.82E-15	1.69E-04	0.235	5.58E-15	1.57E-04
0.276	6.74E-15	2.02E-04	0.266	6.56E-15	1.87E-04	0.256	6.29E-15	1.73E-04
0.182	3.85E-15	1.29E-04	0.168	3.58E-15	1.13E-04	0.168	3.61E-15	1.09E-04

C_22_			C_23_			8Cn		
x_i_	K_G_	α_det_	x_i_	K_G_	α_det_	x_i_	K_G_	α_det_

0.180	4.03E-15	1.13E-04	0.174	3.88E-15	1.05E-04	0.100	4.32E-15	7.04E-05
0.204	4.72E-15	1.30E-04	0.197	4.54E-15	1.20E-04	0.200	5.19E-15	1.46E-04
0.226	5.41E-15	1.45E-04	0.219	5.21E-15	1.35E-04	0.300	6.03E-15	2.26E-04
0.248	6.12E-15	1.61E-04	0.240	5.88E-15	1.49E-04	0.496	6.96E-15	4.02E-04
0.155	3.33E-15	9.63E-05	0.155	3.35E-15	9.28E-05

**Table 14:** Growth rate constant, K_G_ (m/s), and detectable fraction of crystallised volume, α_det_, calculated for each concentration of 8 alkanes and a mixture of 8 alkanes in toluene for the KBHR approach instantaneous nucleation case.

Alkanes in Toluene								
C_16_			C_17_			C_18_		
x_i_	K_G_	α_det_	x_i_	K_G_	α_det_	x_i_	K_G_	α_det_
0.124	4.97E-15	4.89E-05	0.118	4.69E-15	4.41E-05	0.112	4.58E-15	3.95E-05
0.142	5.83E-15	5.66E-05	0.135	5.51E-15	5.10E-05	0.128	5.36E-15	4.57E-05
0.159	6.69E-15	6.42E-05	0.151	6.31E-15	5.79E-05	0.144	6.15E-15	5.19E-05
0.175	7.55E-15	7.16E-05	0.167	7.12E-15	6.47E-05	0.159	6.91E-15	5.80E-05
0.200	8.97E-15	8.35E-05	0.201	9.02E-15	7.98E-05	0.185	8.41E-15	6.91E-05

C_19_			C_20_			C_21_		
x_i_	K_G_	α_det_	x_i_	K_G_	α_det_	x_i_	K_G_	α_det_

0.107	4.36E-15	3.60E-05	0.102	4.24E-15	3.27E-05	0.098	4.05E-15	2.99E-05
0.122	5.11E-15	4.17E-05	0.117	4.97E-15	3.80E-05	0.112	4.75E-15	3.47E-05
0.137	5.85E-15	4.74E-05	0.131	5.70E-15	4.32E-05	0.126	5.45E-15	3.95E-05
0.152	6.60E-15	5.30E-05	0.145	6.44E-15	4.83E-05	0.140	6.15E-15	4.42E-05
0.185	8.45E-15	6.64E-05	0.171	7.84E-15	5.81E-05	0.171	7.87E-15	5.57E-05

C_22_			C_23_			8Cn		
x_i_	K_G_	α_det_	x_i_	K_G_	α_det_	x_i_	K_G_	α_det_

0.094	3.95E-15	2.74E-05	0.090	3.80E-15	2.53E-05	0.100	4.40E-15	3.44E-05
0.107	4.62E-15	3.19E-05	0.103	4.44E-15	2.93E-05	0.199	5.21E-15	7.42E-05
0.121	5.31E-15	3.62E-05	0.116	5.10E-15	3.34E-05	0.297	6.03E-15	1.21E-04
0.134	5.99E-15	4.06E-05	0.129	5.76E-15	3.74E-05	0.500	6.96E-15	2.40E-04
0.158	7.28E-15	4.88E-05	0.158	7.32E-15	4.70E-05			

The growth rate constant K_G_ was calculated using the equation below derived from the spiral growth case from Kashchiev [1];

(1) $$K_{G}\;=\frac{\varepsilon dD_{ef}C\text{kT}}{15\kappa }$$

The sticking coefficient ε was assumed to be 1, the diameter of a solute molecule, d, was calculated from $d=\sqrt[{3}]{6\;\times \nu /\pi }$ where ν is the solute molecular volume, the solute diffusion coefficient *D_ef_*, was assumed to be 10^9^ nm^2^/s [2], C was the concentration in mol nm^−3^. The crystallite growth shape factor, *k_v_*, was taken as *4H_0_* for a square plate crystal, *m* was taken as 1 for growth by diffusion of solute through a stagnant layer around the crystallite, as *m* = ½ applied to the case where growth was controlled by undisturbed diffusion of solute and each solution was magnetically agitated. The dimensionality of crystallite growth, *d,* was taken as 2 for disks or plates. *n* was calculated from the linear fit of $\ln q=\ln q_{0}+\left(n+1\right)\ln u_{c}$.

The detectable fraction of crystallised volume was calculated using:

(2) $$\alpha_{det}=\frac{\left(x_{i}\times \left(MW_{solvent}\frac{1}{\rho_{solvent}}\right)\right)\times \;0.001}{\left(MW_{solute}\frac{1}{\rho_{solute}}\right)-\left(\left(x_{i}\times \left(MW_{solute}\frac{1}{\rho_{solute}}\right)\right)-\left(x_{i}\times \left(MW_{solvent}\frac{1}{\rho_{solvent}}\right)\right)\right)}$$

### 2.4 Multi-homologous mixture composition

Table 15 shows the composition of a model fuels composed of 8 alkanes C_16_H_34_ - C_23_H_48_ in dodecane and toluene as well as a reference diesel fuel. Table 16 shows the provenance of the chemicals used in this study.

**Table 15:** Mole Fraction (x_i_) of alkanes C_16_-C_23_ in a multi-homologous mixture in dodecane and toluene based on the composition of said alkanes in a real diesel fuel.

	Mole Fraction (*x_i_*) of Alkanes		
Alkane	Diesel	Dodecane	Toluene
16	0.173399986	0.173123375	0.172125456
17	0.201569078	0.191359818	0.199465859
18	0.169396885	0.172158059	0.167922309
19	0.194174283	0.197354844	0.197674515
20	0.099177065	0.101012694	0.098199779
21	0.087020856	0.088590479	0.088273402
22	0.047265754	0.047760837	0.048541466
23	0.027996093	0.028639893	0.027797215

**Table 16:** Summary of the raw materials used in this research detailing the nomenclature, chemical formula, provenance, purity and molecular weight.

Name	Formula	Synonym	Purity (wt%)	MW g/mol	Supplier
Hexadecane	C_16_H_34_	C_16_	99	226.45	Sigma Aldrich
Heptadecane	C_17_H_36_	C_17_	99	240.47	Acros Organics
Octadecane	C_18_H_38_	C_18_	99	254.50	Sigma Aldrich
Nonadecane	C_19_H_40_	C_19_	99	268.53	Alfa Aesar
Eicosane	C_20_H_42_	C_20_	99	282.55	Acros Organics
Heneicosane	C_21_H_44_	C_21_	97.5	296.58	Acros Organics
Docosane	C_22_H_46_	C_22_	99	310.61	Sigma Aldrich
Tricosane	C_23_H_48_	C_23_	98	324.63	Acros Organics
Dodecane	C_12_H_26_	C_12_	99	170.34	Acros Organics
Toluene	C_7_H_8_	N/A	99	92.14	Acros Organics

## 3 Experimental Design, Materials and Methods

### 3.1 Materials

*n*-Alkanes, representative of the major constituents of diesel fuels, were chosen for this study, C_16_H_34_ – C_23_-H_48_. Toluene and dodecane were chosen as representative for both the aromatic and aliphatic solvent components of diesel fuels. A model 8-alkane mixture was constructed where the relative composition of C_16_H_34_ – C_23_H_48_ matched that of a real diesel fuel. Table 15 shows the relative composition of the 8-alkane mixture in mole fraction, Table 16 shows the summary of the raw materials used in this research.

### 3.2 Equipment

The polythermal crystallisation experiments were conducted using a Technobis Crystal16 [3] crystallisation screening platform. This is a multicell crystalliser consisting of 16 individual cells which can take a 1.5mL vial of solution. 4 cells are grouped into a block, resulting in 4 blocks which can be individually temperature controlled via a Peltier heat exchanger linked to a refrigerated recirculating bath. The system can cool to ca. -15°C and heat to an upper limit of 100°C at a range of cooling rates from 0.1 - 10°C/min. A dry air purging system prevents condensation when cooling below sub-ambient temperatures. Each cell holds 1mL of solution in which crystallisation and dissolution can be followed as a function of temperature and time by turbidity via a transmission turbidity red laser source/detector, with each cell being magnetically agitated with a 2 × 7mm stirrer bar.

Polythermal crystallisation experiments were also carried out on a Mettler Toledo DSC 1 with cryogenic cooler, for the purpose of determining the thermodynamic parameters of an 8-alkane mixture. Samples were prepared around 10mg in mass in a sealed standard aluminium crucible.

### 3.3 Experimental procedure

#### 3.3.1 Solution preparation

All solutions were prepared on a 5mL scale, as this was a sufficient volume to provide solution to fill 4 Crystal16 cells at once to provide a full run of cooling rates. For each alkane 5 concentrations were prepared, which were unique for each alkane but ranged overall from 0.09 - 0.311 *x_i_*. Solutes were weighed out using a balance of ±0.01mg accuracy. 5mL of solvent was added using a Sartorius 500-5000µL mechanical pipette.

Mixtures were heated on a hotplate with a magnetic stirrer to a temperature high enough that the solution completely dissolved and were left to homogenise at this temperature for 1 hour. A Sartorius 1-1000µL mechanical pipette was used to transfer 1mL of solution to a 1.5mL glass GC vial, in which a 2 × 7mm PTFE stirrer bar had been placed. 16 vials were filled with 4 concentrations in 4 vials each, providing an almost complete set of concentrations to run at 7 cooling rates on the Crystal16

In the case of the 8-alkane mixture pre-determined amounts of each alkane were weighed into a large dish. This mixture was then completely melted over a water-bath, with an inverted watch-glass placed over the mixture so that any condensation formed drained away from the mixture. After homogenising for 1 hour above its melting point, the mixture was weighed into vials and dissolved as described above.

#### 3.3.2 Polythermal analysis

Each block was set to follow a specific temperature program of heating and cooling at prescribed rates so that each concentration would be run at 0.25, 0.5, 1, 2, 3.2, 4 and 5°C/min. 4 blocks allowed 4 cooling rates to be run simultaneously on 4 different solution concentrations. Each temperature program would begin with heating to 40°C, with this temperature being held for 30 minutes to ensure complete dissolution. The sample was then cooled at the specified rate to -15°C, before being held at this temperature for 30 minutes to ensure the system stabilised, before heating back up at the same rate to 40°C. This cycle was repeated 4 times. From this the crystallisation temperature and dissolution temperature were determined, by fitting a Boltzmann [4] curve to the transition %T vs. temperature data and determining the temperature at 99%T for T_diss_ and 90%T for T_cryst_ from the equation of the fit.

This data was then calibrated based on the linear response of the temperature of a thermocouple inside a cell containing only solvent, and the Crystal16 measured temperature for each block. With the correct block's calibration being applied to the data that was collected on that block and with that solvent.

This data was then analysed using the polythermal method of plotting T_c_ and T_d_ vs cooling rate to determine the equilibrium values at *q*=0°C/min, T_c,l_ and T_e_. The solubility was modelled with the Van't Hoff equation along with the solute's ideal solubility. The nucleation mechanism and associated kinetic parameters were determined using the KBHR approach [5], [6], [7], a complete walk through of this approach can be found here [8].

The nucleation mechanism was determined from the KBHR approach using the “rule of 3”. For a plot of ln(*q*) vs. ln(*u_c_*), where *q* is the cooling rate (Ks^-1^) and *u_c_* is the relative critical undercooling as defined by;

(3) $$\ln\left(u_{c}\right)=\ln\left(\frac{T_{e}-T_{c}}{T_{e}}\right)$$

The gradient of a linear fitting of this plot, known as the nucleation mechanism parameter ω, determines the mechanism. When ω > 3 the mechanism in progressive and when ω < 3 the mechanism is instantaneous.

For the progressive case the critical cluster radius r*, number of molecules in the critical cluster i*, and the effective interfacial tension *γ_eff_* can be determined by fitting Eq. 4 to a graph of ln(*q*) vs. ln(*u_c_*).

(4) $$\ln\left(q\right)=\ln\left(q_{0}\right)+a_{1}\ln\left(u_{c}\right)-\frac{a_{2}}{\left(1-u_{c}\right)u_{c}^{2}}$$

The parameters *a_1_, a_2_* and *q_0_* are given by.

(5) $$a_{1}=3$$

(6) $$a_{2}=b$$

(7) $$q_{0}=\frac{VK_{J}T_{e}}{N_{det}2b}$$

Where V is the solution volume, K_J_ is the nucleation rate constant and N_det_ is the number of crystallites at the detection point. From the parameter a_2_ γ_eff_ can be determined through.

(8) $$b=\frac{k_{n}v_{0}^{2}\gamma_{eff}^{3}}{kT_{e}\lambda^{2}}$$

k_n_ is the nucleus shape factor and v_0_ is the volume occupied by a solute molecule in the crystal. r* and i* can then be calculated using.

(9) $$r^{*}=\frac{2\gamma_{eff}v_{0}}{\lambda u^{3}}$$

(10) $$i^{*}=\frac{2bkT_{e}}{\lambda u^{3}}$$

For the instantaneous case the concentration of instantaneously nucleated crystallites *C_0_* can be determined by fitting Eq. 11 to a graph of of ln(*q*) vs. ln(*u_c_*).

(11) $$\ln\left(q\right)=\ln\left(q_{0}\right)+\left(n+1\right)\text{ln}\left(u_{c}\right)$$

C_0_ can then be determined from.

(12) $$q_{0}={\left[\frac{k_{v}C_{0}}{{\left(n+1\right)}^{d}\alpha_{det}}\right]}^{1/md}a^{n}K_{G}T_{e}$$

Where k_v_ is the crystallite growth shape factor $\frac{4\pi }{3}$ for spheres, 8 for cubes, πH_0_ for disks, 4H_0_ for square plates (H_0_ is the fixed disk or plate thickness), and 2A_0_ for needles (A_0_ is the fixed needle cross-sectional area). K_G_ is the growth rate constant. d is the dimensionality of crystallite growth, 3 for spheres or cubes, 2 for disks or plates and 1 for needles. a is the dimensionless molecular latent heat of crystallization and is defined in Eq. 13 as:

(13) $$a=\frac{\lambda }{kT_{e}}$$

Where λ is the latent heat of crystallization and k is the Boltzmann constant. α_det_ is the detectable fraction of crystallized volume.

#### 3.3.3 Computational methods

Intermolecular interactions were determined using dedicated software [9], [10], [11] that uses grid-based modelling with a systematic search algorithm to investigate the intermolecular interactions between a target and probe molecule by calculating the interaction energy between the two at each point on a user defined grid. The molecules were constructed and optimised using the Forcite module of Materials Studio [12] before being run in the grid search.

For both solvents the optimal grid was determined by establishing the number and energy of interactions found as a function of grid size. Ideally a grid with infinitely high resolution would be used however software and hardware limited the total number of grid points to less than ≈1000. As a result, the optimal spacing of these grid points was determined to find the strongest interactions. In the case of Toluene this was a 30-30-30 Å (XYZ) size grid with 8-11-8 (UVW) and 972 grid points, for dodecane this was 20-20-30 Å (XYZ) Å size grid with 7-11-8 (UVW) grid lines. The Euler angles in all cases were 30° at all grid points. XYZ represents the physical size of the grid in angstroms, UVW represents the number of grid lines in a specific direction, XYZ, from the origin.

The interaction energy at each of the individual grid points and Euler angles from these optimised grids, was calculated for each *n*-alkane target and solvent probe pair. From the interaction energies of all interactions for a given probe/target pair, the strength of the strongest interaction was determined, as well as the total number of interactions with values above a threshold value e.g., -1.0kCal/mole, and the total sum of energies of all the interactions above these threshold values.

## Ethics Statement

The authors confirm that this work meets the ethical requirements of Data in Brief.

## CRediT authorship contribution statement

**Alexander S.M. Jackson:** Conceptualization, Methodology, Investigation, Formal analysis, Writing – original draft, Writing – review & editing. **Dhanesh Goberdhan:** Supervision, Resources, Writing – review & editing. **Peter J. Dowding:** Supervision, Resources, Writing – review & editing. **Kevin J. Roberts:** Conceptualization, Supervision, Writing – review & editing, Funding acquisition.

## Declaration of Competing Interest

The authors declare that they have no known competing financial interests or personal relationships that could have appeared to influence the work reported in this paper.

## Data Availability

Data for Crystallisation of a Homologous Series of Single and Mixed n-Alkanes (C16 – C23) from Representative Hydrocarbon Fuel Solvents (Original data) (Research Data Leeds Repository).
